# The community-based delivery of an innovative neonatal kit to save newborn lives in rural Pakistan: design of a cluster randomized trial

**DOI:** 10.1186/1471-2393-14-315

**Published:** 2014-09-08

**Authors:** Ali Turab, Lisa G Pell, Diego G Bassani, Sajid Soofi, Shabina Ariff, Zulfiqar A Bhutta, Shaun K Morris

**Affiliations:** Department of Paediatrics and Child Health, Division of Women and Child Health, Aga Khan University, Karachi, Pakistan; Centre for Global Child Health, The Hospital for Sick Children, Toronto, Canada; Center of Excellence in Women and Child Health, Aga Khan University, Karachi, Pakistan; Division of Infectious Diseases, The Hospital for Sick Children, Toronto, Canada; Department of Paediatrics, Faculty of Medicine, University of Toronto, Toronto, Canada

**Keywords:** Neonatal mortality, Pakistan, Lady health workers

## Abstract

**Background:**

Worldwide, an estimated 2.9 million neonatal deaths occurred in 2012, accounting for 44% of all under-five deaths. In Pakistan, more than 200,000 newborns die annually and neonatal mortality rates are higher than in any other South Asian country and haven’t changed over the last three decades. The high number of neonatal deaths highlights the urgent need for effective and sustainable interventions that target newborn mortality in Pakistan.

**Method/Design:**

This cluster randomized trial aims at evaluating the impact of delivering an integrated neonatal kit to pregnant women during the third trimester of pregnancy and providing education on how to use the contents (intervention arm) compared to the current standard of care (control arm) in the district of Rahimyar Khan, Punjab province, Pakistan. The kit, which will be distributed through the national Lady Health Worker program, comprises a clean delivery kit (sterile blade, cord clamp, clean plastic sheet, surgical gloves and hand soap), sunflower oil emollient, chlorhexidine, ThermoSpot™, Mylar infant sleeve, and a reusable instant heat pack. Lady health workers will be provided with a standard portable hand-held electric weighing scale. The primary outcome measure is neonatal mortality (death in the first 28 days of life).

**Discussion:**

While many cost-effective, evidence-based interventions to save newborn lives exist, they are not always accessible nor have they been integrated into a portable kit designed for home-based implementation entirely by caregivers. The implementation of cost-effective, portable, and easy-to-use interventions has tremendous potential for sustainably reducing neonatal mortality and long-term improvements in population health. The bundling of interventions and commodities together also has much potential for cost-effective delivery and maximizing gains from points of contact. This study will provide empirical evidence on the feasibility and effectiveness of the delivery of an innovative neonatal kit to pregnant women in Pakistan. Together, these findings will help inform policy on the most appropriate interventions to improve newborn survival.

**Trial registration:**

ClinicalTrial.gov NCT02130856. Registered May 1, 2014.

## Background

### Neonatal mortality worldwide and in Pakistan

Globally, 2.9 million newborns died in 2012 within their first month of life, accounting for approximately 44% of all under-five deaths [1]. While global newborn mortality is declining, the achievements among neonates (0 – 28 days of life) are dismal compared to the reductions in post-neonatal deaths for both infants (1-11 months of age) and children (1 to 4 years of age).

Most neonatal deaths are largely preventable and attributable to preterm birth complications (34%), intrapartum-related complications (25%), and infectious causes (22%) such as sepsis, meningitis and pneumonia [2]. Notably, many of these major causes of newborn death, namely infection and hypothermia, also negatively impact development of the growing brain. It is therefore not surprising that more than 200 million children under 5 years, almost all in low- and middle-income countries (LMICs), are not reaching their developmental potential [3].

Marked disparities in neonatal mortality rates (NMRs) exist between countries and regions. The majority of neonatal deaths occur in low-income and middle-income countries with the highest rates in sub-Saharan Africa and South Asia [1, 4]. Strikingly, more than half of all newborn deaths are attributable to only five countries: India, Nigeria, Pakistan, China, and the Democratic Republic of Congo [5].

In Pakistan, more than 200,000 newborns die annually and in 2012, an estimated 42 newborns per every 1000 live births died during the neonatal period [1]. Demographic and Health Surveys collected in Pakistan between 1990 and 2012 demonstrate that neonatal mortality has increased in every province except Khyber Pakhtunkhwa [6]. Notably, in Punjab province neonatal mortality has increased from 58 to 63 deaths per 1000 live births [6]. In addition, within Pakistan the risk of neonatal death is higher in rural areas than in urban areas, in the lowest wealth quintile than in the highest wealth quintile, and to mothers with no education than to mothers with higher education [6]. In rural Pakistan, most newborn deliveries occur at home with minimal supervision from skilled birth attendants. The problem is further compounded by the frequent adoption of unsafe newborn care practices including the use of unsterilized instruments to cut the umbilical cord, the application of unsafe substances to the umbilical stump [7], and taboos preventing early uptake of best practices such as feeding of colostrum and early initiation of breastfeeding. Together, these newborn care practices are associated with increased risk of neonatal sepsis and mortality [8].

### Primary health care infrastructure in Pakistan

The public health care delivery infrastructure in Pakistan is three-tiered and includes primary, secondary and tertiary care facilities [9]. Primary health care (PHC) infrastructure in Pakistan comprises over 13,000 first-level care facilities including a network of rural health centers (RHCs), basic health units (BHUs), dispensaries, and maternal and child health centres (MCHC) [9]. Since over 60% of the Pakistan population resides in rural areas [10], health care facilities are not always accessible and thus, community-level care is supported by trained community health workers (CHWs), known as Lady Health Workers (LHWs). The Lady Health Worker program was launched by the government of Pakistan in 1994 and aims to provide PHC services to communities in rural areas and urban slums [11]. Specifically, the program targets unmet health needs in the area of maternal, newborn, child and reproductive health and provides family planning services, and integrates with other vertical health programs. LHWs are each affiliated with a government health facility (BHU) and form an essential link between the formal health system and the community. LHWs each serve a population of approximately 210 households. LHWs are not the primary earner of their family and receive a small monetary allowance of approximately $840 USD per year. They are provided with basic medical supplies that includes essential drugs for the treatment of minor conditions such as diarrhea and acute lower respiratory infection (ALRI) as well as contraceptives for eligible couples [11]. Currently, over 100,000 LHWs liaise between the formal health system and communities to provide antenatal care, contraceptive advice, newborn growth monitoring, immunization services, and health education on hygiene and sanitation [11].

### Rationale and contents of integrated neonatal kit

The high neonatal mortality rates in Pakistan require urgent global attention. While many proven, cost-effective ways to save newborn lives exist, they are not always accessible nor have they been integrated into a single portable kit for standardized home-based caregiver-implementation and advocacy. Such a kit consisting of low-cost, evidence-based interventions has potential to reduce neonatal mortality.

**Figure 1:**
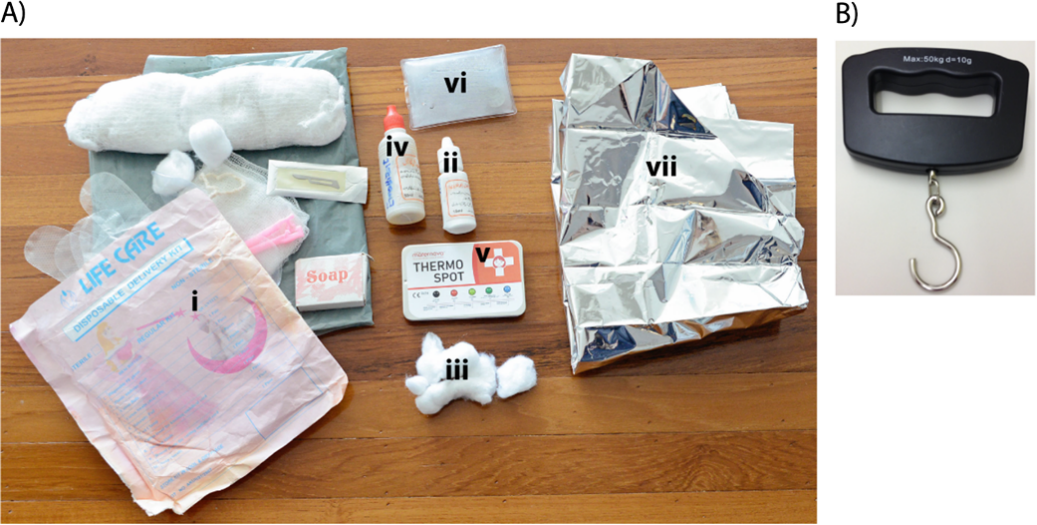
**Integrated neonatal kit contents. A)** The neonatal kit includes a i) clean delivery kit, ii) 4% chlorhexidine solution that is to be applied with iii) cotton balls, iv) sunflower oil emollient, v) ThermoSpot, vi) a reusable instant heat pack, and vii) a Mylar infant blanket. **B)** A handheld battery operated weighing scale will be provided to LHWs in the intervention cluster.

In this cluster-randomized controlled trial, several interventions and commodities that have individually been shown to either reduce the incidence of neonatal insults or provide early identification of danger signs have been integrated into a low-cost and portable neonatal kit. The kit comprises a clean birth kit (plastic sheet, cord clamps, sterile blade, gloves, and soap), 4% chlorhexidine solution, sunflower oil emollient, a temperature monitoring device called ThermoSpot™, a Mylar infant warming blanket, and a reusable, instant heat pack that produces heat through an exothermic crystallization reaction of a non-toxic salt-based solution. LHWs will also be equipped with a hand-held electric weighing scale to identify low birth weight (LBW) newborns. Table 1 provides a detailed description of the contents (Figure 1), utilization and evidence surrounding each kit component. Importantly, while the efficacy of most kit components have been validated in isolation, their combined impact remains unclear. In addition, the effectiveness of several kit components, when implemented in community-based settings and delivered entirely by non-health workers remains to be evaluated.

**Table 1:** **Neonatal kit components, utilization and evidence**

Kit component	Description	Utilization	Evidence
**Clean birth kit (CBK)**	● Contains a sterile blade to cut the umbilical cord, clean plastic square to be placed on the ground or other surface during birth, plastic gloves to be worn by the birth attendant, hand soap, and cord ties/clamp	● The expectant mother will be taught to provide the CBK to the traditional birth attendant (TBA) or other individual conducting the delivery	● Clean birth kits, when combined with education, are associated with reductions in newborn mortality and infection [12]
		● Expectant mothers will be instructed to take the CBK to health facilities where it may be used at the discretion of the health professional conducting the delivery	
**Chlorhexidine (CHX) solution**	● Each kit will contain 15 mL of 4% CHX packaged into a small plastic dispensing bottle and accompanied by a bag of cotton balls	● Caregivers will be taught to apply a small amount of CHX to a cotton ball and to dab the wet cotton ball onto the umbilical stump and surrounding area once a day from day 1 to day 10 of the newborn’s life	● CHX is a topical antiseptic that exhibits broad-spectrum antimicrobial activity against both Gram-positive and Gram-negative bacterium
			● Umbilical-cord cleansing with CHX solution reduces the incidence of omphalitis and neonatal mortality [13–16]
**Sunflower oil emollient**	● Each kit will contain 50 mL of sunflower oil emollient packaged into a small plastic dispensing bottle	● Caregivers will be taught to take a small amount of oil into their hands and massage the oil into the newborn’s skin all over their body once a day from day 3 until day 28 of the newborn’s life	● In facility-based studies, sunflower oil has been shown to protect against hospital-acquired infections in very low birth weight infants [17, 18] and improve survival rates in hospitalized preterm infants [19]
Mechanically extracted from raw sunflower seeds using an expeller press			
**ThermoSpot** ^**TM**^	● ThermoSpot^TM^ is a small circular device ~12 mm in diameter that adheres to skin and allows for non-invasive, continuous thermal monitoring [20]	● Caregivers will be taught to apply one sticker to the axillary region of the newborn on their first day of life and to leave the sticker in place until it falls off on its own or until day 14 of life (if sticker falls off it will be replaced)	● Today, the acceptability and accuracy of ThermoSpot^TM^ has been demonstrated in both hospital [21–23] and community-based settings [24] within developing countries
ThermoSpot^TM^ was obtained from Maternova (http://maternova.net)			
	● ThermoSpot^TM^ changes colour as temperature changes [25]		
	● Device adheres to the skin for ~24 days [22]	● Caregivers will be taught that a bright green face (newborn temperature 36.5°C to 37.5°C) is normal; a blue face (temp >39.0°C) indicates fever and care should be immediately sought at a local health facility; a pale green and red faces (temp 35°C to 36°C) indicate moderate hypothermia; and, a black face (temp < 35°C) represents severe hypothermia	
		● In the event of a pale green face, the caregiver will be instructed to initiate kangaroo care (hold the baby close to her skin), and frequently monitor temperature	
		● In cases of moderate to severe hypothermia (red and black ThermoSpot), caregivers will be taught to place the infant in the Mylar sleeve with warmer and immediately take the newborn to the local health centre	
**Mylar infant sleeve**	● One sterile Mylar infant sleeve will be included in each kit	● If ThermoSpot^TM^ indicates moderate or severe hypothermia, caregivers will be taught to activate the non-electric warmer and place it at the bottom of the Mylar sleeve and then the newborn is to be placed in the sleeve	
Mylar infant sleeves were obtained from Maternova (http://maternova.net)			
		● Once the newborn is in the Mylar sleeve, the caregiver is to immediately take the child to the nearest health facility for further management	
**Reusable instant heat pack**	● Instant heat packs are pocket-sized, reusable, and placed in a fitted cloth pouch	● If ThermoSpot^TM^ indicates moderate or severe hypothermia, the instant heat pack will be activated and placed, while still inside cloth pouch, at the base of Mylar infant sleeve	
	● Each heat pack contains a non-toxic salt-based solution and a small metallic disc		
	● By flexing the metallic disc, an exothermic crystallization reaction is triggered, which keeps the temperature warm for ~20 to 30 minutes		
**Handheld battery operated weighing scale with suspended cloth sling**	● Scale (Shenzhen Cyber Technology Ltd. Item # CT8831) will not be included with the kit but rather one will be issued to each LHW in the intervention cluster	● The LHW will be instructed to weigh the newborn at the first home visit after delivery	
		● The newborn will be weighed twice and the average of these two weights will be recorded as the birth weight

### Hypothesis

We hypothesize that delivery of the neonatal kit to pregnant women during their third trimester of pregnancy and the provision of education on how to use the individual kit components will increase coverage rates of essential interventions and reduce NMR by approximately 40% through a reduction in both infectious causes of death and those associated with the consequences of prematurity and LBW.

## Methods/Design

### Study setting

**Figure 2:**
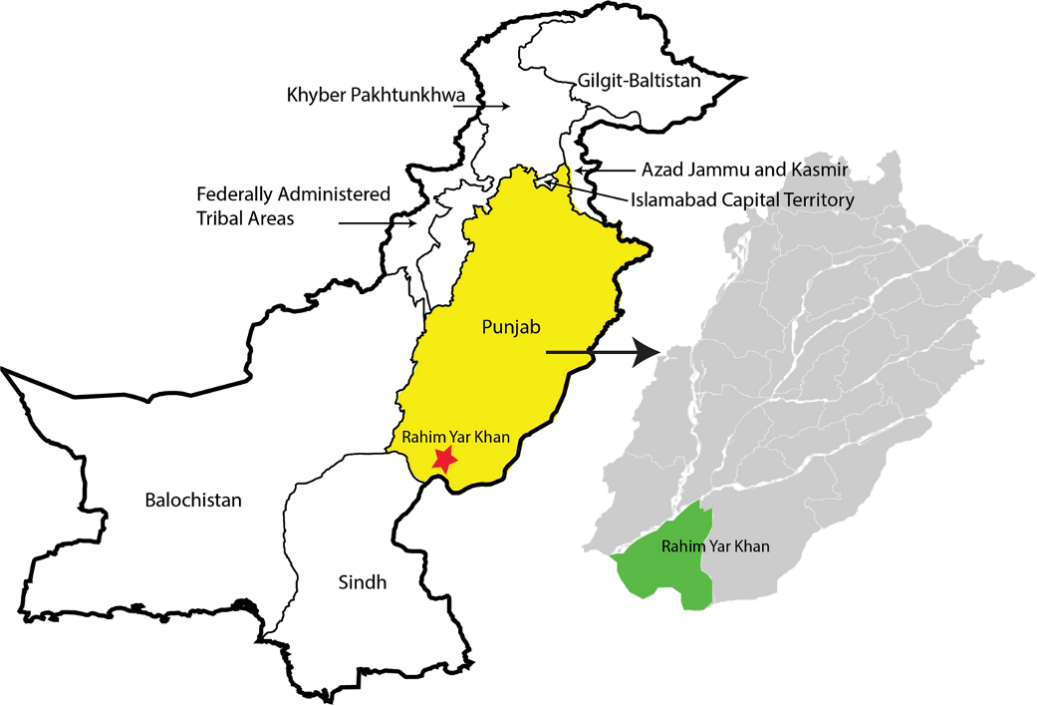
**Map of Pakistan showing the study area in District Rahimyar Khan, Punjab province.**

This study will be conducted in district Rahimyar Khan (RYK) in Punjab province, Pakistan (Figure 2). The country has several distinct geographical regions, including the mountainous north and northwest, the flat plain of river Indus in the east, and Balochistan plateau in the west. RYK, which is located in the flat plain of river Indus, spans a geographical area of 11,880 square kilometers, making it the fourth largest district in Punjab. Most of the country experiences extreme seasonal variations in temperature, which range from below 0°C during winter months and highs of 40°C during the summer. There are 40 Union Councils in district RYK, which are small geographical subdivisions defined for administrative purposes. Union Councils each serve between 20,000 to 40,000 people and typically contain a BHU or RHC that provides basic maternal and newborn care. Union Councils are further subdivided into smaller geographical areas called principal villages. One or more LHW is assigned to each principal village where they are each responsible for the provision of PHC services to approximately 210 households.

In 2012, the estimated population of RYK was 4.5 million people with a density of 385 individuals per square mile. Currently, the estimated birth rate is 25 per 1000 population and approximately 55% of all births in the district take place at home. The NMR in RYK is 42 per 1000 live births.

### Study population

All pregnant women and their home- or facility-born live births within participating clusters will be eligible for enrollment in this study.

### Study design, cluster definition and sample size

**Figure 3:**
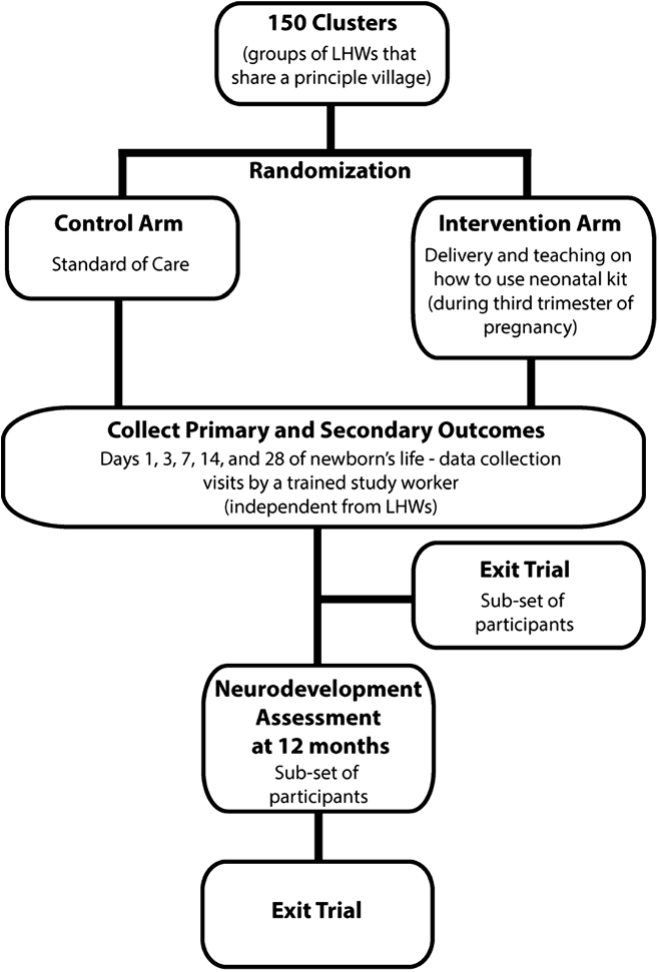
**Schematic diagram of trial activities.**

This study is designed as a cluster randomized, pragmatic, open label controlled intervention trial that will be conducted within the existing health care infrastructure in Pakistan. Specifically, both the intervention (neonatal kit) and control (standard care) will be delivered to participants by LHWs (Figure 3). A cluster has been defined as a principal village and its associated LHWs.

With an estimated live birthrate of 25 per 1000 population, baseline NMR of 42 per 1000 live births, and a population of ~35 live births per cluster, the randomization of 150 clusters, half to intervention and half to control, with 90% enrollment of eligible mothers and including up to a 10% loss to follow up will provide >80% power to detect a 40% reduction in mortality in the intervention compared to the control arm. All eligible women within a cluster will receive the same intervention. Since clusters are geographically separated from one another and families within each cluster are typically non-mobile, contamination is unlikely but to further avoid contamination, unused kit components will be collected from participants during their last data collection visit (day 28 of newborn’s life). Unused kit components will be also be collected in cases of stillbirths and child death after enrollment.

### Randomization

Randomization will be performed on principal villages (clusters) stratified into two groups before randomization: villages with 1 LHW and those with more than 1 LHW. To balance cluster size, one large village will be excluded and the remaining villages will be randomized into two groups (intervention and control). To ensure that randomization is unbiased, a scientist who is not directly involved in the research project will perform the cluster-stratified randomization.

### Delivery of the integrated neonatal kit

To facilitate future scale-up efforts, the delivery of the neonatal kit will be built into the existing schedule of LHW home visits. As part of the current program, LHWs routinely visit pregnant women during their third trimester of pregnancy. During the third trimester visit, following a study worker obtaining consent, LHWs will leave the kit with all enrolled pregnant women in intervention clusters and will teach caregivers how to properly use the components of the kit. There will not be any additional health education component beyond that pertaining to the use of the kit contents. In order to optimize data collection in the critical period immediately following delivery, a small incentive will be provided to the first person that notifies the study team of a birth within 24 hours of delivery. To facilitate birth notifications, LHWs, community leaders, and the family of expectant women will be provided with an information card outlining instructions to call a specific phone number within 24 hours of delivery. The phone call will be directed to a study research officer who will maintain a daily call log and disburse the incentive to the first party who provided birth notification. In the existing LHW program, LHWs are meant to visit all births within 24 hours of delivery and thus, at this post-delivery visit, LHWs will reiterate their teachings regarding the correct use of the neonatal kit contents.

### Study outcome measures

The primary outcome in this study is all-cause neonatal mortality. Secondary outcomes include the incidence of omphalitis, severe infection, identified cases of hypothermia and hyperthermia, number of LBW newborns identified, health facility usage, and neurodevelopment measured at 12 months of age (Table 2). Due to the nature of the intervention, blinding is not possible, however to reduce measurement bias, outcome data will be collected by an independent team that will not be involved in the delivery of the intervention.

**Table 2:** **Primary and secondary outcome measures**

Outcome	Definition
**Primary outcome**	
All-cause neonatal mortality	Death from any cause in the first 28 days of life
**Secondary outcomes**	
Incidence of omphalitis	A) None (no redness or swelling);
	B) Mild (inflammation limited to the cord stump);
	C) Moderate (inflammation extending to the skin at the base of the cord stump less than 2 cm); or
	D) Severe (inflammation extending more than 2 cm from the cord stump)
Incidence of severe infection	A) Convulsions; or
	B) Fast breathing (60 breaths per minute or more); or
	C) Severe chest indrawing; or
	D) Movement only when stimulated or no movement at all; or
	E) Not feeding at all for at least 12 hours
Cases of hypothermia and hyperthermia identified	Defined using ThermoSpot:
	A) Moderate hypothermia: pale green and red face (35°C to 36°C)
	B) Severe hypothermia: black face (<35°C)
	C) Hyperthermia: blue face (>39°C)
Number of LBW newborns identified	<2500 grams at first weighing
Health facility use	Health centre visits to
	A) Dispensary
	B) Basic Health Unit (BHU)
	C) Rural Health Centre (RHC)
	D) Tehsil Head Quarter (THQ)
	E) District Head Quarter (DHQ)
	F) Private clinic
	G) Private Hospital
Neurodevelopment at 12 months of age	Defined by the neurodevelopment score assigned at 12 months of age as measured by the standardized Bayley Scales of Infant Development III assessment tool

### Contents and utilization of the neonatal kit

The integrated neonatal kit comprises a clean birth kit, 4% chlorhexidine solution, sunflower oil emollient, ThermoSpot™, Mylar infant sleeve, and a reusable, instant heat pack (Figure 1A). In addition, LHWs will be supplied with a handheld electric weighing scale (Figure 1B) with suspended cloth sling to weigh newborns. A detailed description of each kit component, its intended utilization and associated evidence is outlined in Table 1.

### Clean birth kit

Expectant mothers will be taught to provide the clean birth kit to the traditional birth attendant (TBA) or other individual conducting the delivery. If the expectant mother will be delivering her baby in a health facility, she will be instructed to take the clean birth kit to the facility where it may be used at the discretion of the health professional conducting the delivery.

### Chlorhexidine solution (4%)

Caregivers will be taught to apply a small amount of 4% chlorhexidine to a cotton ball and to dab the wet cotton ball onto the umbilical stump and surrounding area once a day from day 1 to day 10 of the newborn’s life.

### Sunflower oil emollient

The caregiver will be taught to take a small amount of oil into their hands and massage the oil into the newborn’s skin all over their body once a day from day 3 until day 28 of the newborn’s life. In this study, sunflower oil was mechanically extracted from raw sunflower seeds using an expeller press.

### ThermoSpot™

ThermoSpot™ is a small circular sticker that adheres to the skin and changes colour as temperature changes [25], allowing for continuous temperature monitoring. Five ThermoSpot™ stickers will be provided in each kit. Caregivers will be taught to apply one sticker to the axillary region of the newborn on their first day of life and to leave the sticker in place until it falls off on its own or until day 14 of life. If a sticker falls off, it will be replaced with a new one. The meaning of the appearance of each ThermoSpot™ will be explained to caregivers as well as the measures to be taken if the device indicates any sign of fever or hypothermia.

### Mylar infant sleeve

The caregiver will be taught that if ThermoSpot™ indicates moderate or severe hypothermia (Table 1), the non-electric warmer is to be activated and placed at the bottom of the Mylar sleeve and then the newborn is to be placed in the sleeve. Once the newborn is in the Mylar sleeve, the caregiver is to immediately take the child to the nearest health facility for further management.

### Reusable instant heat pack

If ThermoSpot™ indicates moderate or severe hypothermia, caregivers will be taught to activate the FDA-approved reusable instant heat pack and place it first inside a cloth pouch, and then at the base of Mylar infant sleeve. The activated heat pack holds its temperature for approximately 20 to 30 minutes.

### Handheld battery-operated weighing scale with suspended cloth sling

The scale will not be included with the kit but rather, one will be issued to each LHW in the intervention cluster. The LHW will be instructed to weigh the newborn at the first home visit after delivery. The newborn will be weighed twice and the average of these two weights will be recorded as the birth weight.

### Data collection and data management

Data collectors will be independent from LHWs and will be selected based on their high level of performance in previous work at the same study site. All data collectors will undergo three days of formal training where they will be taught the goals of the study, basic methodology, and provided with an in-depth understanding of all data collection forms. During training, data collectors will be required to conduct an observed interaction with a mother and newborn in the community to ensure standardization and quality data collection.

Data collectors will visit homes in the intervention and control clusters on day 1 (or as soon as possible after birth notification), 3, 7, 14, and 28 of life. To facilitate birth notifications, LHWs, community leaders, and the family of expectant women will be provided with instructions to call a study research officer within 24 hours of delivery. The research officer will notify the appropriate data collector. At the first visit, the data collector will administer a questionnaire to document the events surrounding delivery and the immediate post-natal status of the newborn and weigh the newborn. At each subsequent visit, the data collector will assess the newborn for primary and secondary outcomes through the administration of a questionnaire covering the events that took place since the last visit. Data collectors will be trained to identify danger signs and will refer the newborn to the LHW, triggering the appropriate cascade of events leading to referral to the most appropriate health facility. Data collectors will not be trained to treat outcome conditions. At any of these visits, the data collectors may be notified of the death of a baby. In the event of a newborn death, the parent will be given the choice of whether or not a verbal autopsy [26] can be performed. At 12 months of age, a proportion (approximately 450) of randomly selected children from each arm of the study will undergo a standardized assessment of neurodevelopment by a study worker trained in the application of the Bayley Scale of Infant Development III (BSIDIII) [27]. The data collector will be blinded to the arm of the trial to which the participant was randomized. The assessment takes up to 1 hour to administer, will be conducted in the homes of participants, and will be performed in the presence of a parent or caregiver. During the BSID standardized test, five key developmental domains of cognition, language, social-emotional, and motor and adaptive behaviour of the child are assessed [27].

Paper data collection forms will be delivered to the data management unit (DMU) on a daily basis where they will be entered in duplicate into electronic format. Prior to data entry, all forms will be checked for completeness and consistency and coded. In cases of inconsistency or missing responses, editors will flag the errors/omissions and consult data collectors for possible explanations. Research officers and supervisors will validate 5% and 2% respectively, of all home visits by recollecting data and comparing data collection forms with those filled in by data collectors. In cases where discrepancies exceed 20%, data collectors will be retrained. Databases will be developed using Microsoft FoxPro and will employ a range of consistency checks to minimize data entry errors.

### Data analysis plan

The primary outcome (death during the neonatal period) will be measured in both the intervention and control arms on days 1, 3, 7, 14, and 28 of a newborn’s life. This trial’s data will be analyzed as intention to treat using univariate and multivariate methods. Risk estimates will be adjusted for cluster allocation via generalized estimating equations using a log link between the binary response variable (binomial family) and the linear predictor, and unstructured correlations between observations. STATA version 13 will be used for all data analyses. Causes of death will be determined by physician review of verbal autopsy. Results will be presented in accordance with CONSORT guidelines.

### Ethical considerations

This study has gained ethical approval from the National Bioethics Committee of the Government of Pakistan, and the Research Ethics Board at The Hospital for Sick Children, Toronto, Canada.

## Discussion

Today, 44% of all under-five deaths occur in the neonatal period (within the first 28 days of life) [1] and progress in newborn survival lags behind the achievements made in maternal and child mortality. For example, while the number of global neonatal deaths has decreased from an estimated 4.6 million in 1990 to 2.9 million in 2012, the number of newborn deaths as a proportion of overall child mortality has increased during this same time period from 37% to 44% [1]. Without increased global action, it is estimated that by 2035 there will be an additional 49 million neonatal deaths [5].

More than half of all neonatal deaths occur in only five countries: India, Nigeria, Pakistan, China, Democratic Republic of Congo [5]. In Pakistan, more than 200,000 newborns die each year and the NMRs are among the highest in the world [1]. In rural Pakistan, most newborns are delivered at home and receive minimal monitoring from skilled health care professionals. Compounding these sub-optimal conditions, unsafe newborn care practices, which are associated with increased risk of neonatal sepsis and mortality [8], are commonly practiced behaviors in the region [7].

While evidence exists in support of various health interventions for reducing neonatal mortality, these interventions are not always accessible or affordable to those who need them most. In resource poor, rural settings, many births occur at home without supervision from medically trained personnel. In these settings, a multitude of interconnected factors such as geography, infrastructure, and poverty act as barriers to high-quality health care. Transformations to the Pakistan health system, including the introduction of the LHW program, place the country in an ideal position to improve population health coverage and decrease neonatal mortality. The LHW program aims to achieve universal health coverage through the provision of PHC care services in rural communities and urban slums. Indeed, knowledge of LHWs among women in rural communities is high. In Punjab province, 84.4% of married women between the ages of 15-49 living in a rural area know about the presence of LHWs in their area, compared to 65.9% in urban areas [6]. Evidence suggests that the empowerment of local community workers in the delivery of health interventions and home-based care leads to a dramatic increase in child survival [28].

In this study, a portable, low-cost, integrated kit comprised of evidence-based interventions will be delivered to pregnant women in RYK, Pakistan through the existing LHW infrastructure. The delivery of the intervention through the LHW program, combined with educating caregivers on how to use the neonatal kit contents will facilitate long-term sustainability. The findings of this trial will provide empirical evidence to inform policy on the implementation of sustainable interventions in rural settings to improve neonatal mortality in Pakistan.
